# Opportunities and Challenges of Establishing a Regional Bio‐based Polylactic Acid Supply Chain

**DOI:** 10.1002/gch2.202200218

**Published:** 2023-05-05

**Authors:** Ali Abdelshafy, Alina Hermann, Sonja Herres‐Pawlis, Grit Walther

**Affiliations:** ^1^ RWTH Aachen University Operation Management 52072 Kackertstr. 7 Aachen Germany; ^2^ RWTH Aachen University Institute for Inorganic Chemistry 52074 Landoltweg 1a Aachen Germany; ^3^ Bioeconomy Science Center (BioSC) Aachen Germany

**Keywords:** bio‐based plastics, circular bioeconomy, polylactic acid, sustainable products

## Abstract

Polylactic acid (PLA) is the bioplastic with the highest market share. However, it is mainly produced from first‐generation feedstock and there are various inconsistencies in the literature in terms of its production and recycling processes, carbon footprint, and prices. The aim of this study is to compile and contrast these aspects and investigate second‐generation PLA production from technical, economic, and ecological perspectives simultaneously. The comprehensive analyses also show the chances and challenges of originating a PLA supply chain in a specific region. Herein, the German Federal State of North Rhine‐Westphalia (NRW) has been chosen as a region of interest. In addition to highlighting the industrial capabilities and synergies, the study quantifies and illustrates the locations of different suitable second‐generation feedstocks in the region. However, the identified potentials can be challenged by various obstacles such as the high demand of bioresources, feedstock quality, spatial aspects, and logistics. Furthermore, the substantial price gap between PLA and fossil‐based plastics can also discourage the investors to include PLA on their portfolios. Thus, the study also provides recommendations to overcome these obstacles and promote the regional value chains of bioplastics which may serve as prototype for other regions.

## Graphical abstract

The study starts with introducing the main differences between the fossil and bio‐based value chains and the main technical and environmental aspects of polylactic acid (PLA) production. Thereafter, the study focuses on investigating the potentials and challenges associated with establishing a regional PLA supply chain (e.g., raw materials and prices). Finally, the main outcomes and conclusions are highlighted.

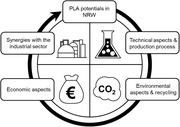

## 1 Introduction

Plastics are very important materials used in all aspects of our daily life and industrial applications. Global plastics production has exceeded 365 Mt in 2020, of which 15% are produced in Europe.^[^ ^1^ ^]^ However, producing fossil‐based plastics requires significant amounts of non‐renewable raw materials, generate significant amounts of GHG emissions (5 ton CO_2_ per ton plastic) (including end‐of‐life treatment).^[^ ^2^ ^]^ Thus, against the background of the global transformation toward sustainability, bioplastics have gained a lot of attention owing to their low carbon foot‐print, sustainable raw materials, and biodegradability. In the recent years, various bioplastics have entered the market (e.g., polyhydroxyalkanoates (PHA) and polylactic acid (PLA)),^[^ ^3^, ^4^, ^5^, ^6^ ^]^ which have been mainly produced from first‐generation feedstocks.

Nonetheless, the limited available land triggers many critics regarding the competition of plastics from first‐generation feedstock with food. Although, the land use to grow feedstock of bioplastics is eventually less than 0.02% of the global agriculture land and so far no competition with land used for food exists,^[^ ^7^ ^]^ some authors expect that using crops to produce bioplastics will not have a significant positive effect on climate change and sustainability, as it may lead for instance to increasing the deforestation rate.^[^ ^8^ ^]^ Therefore, the industrial and scientific effort is moving from the utilization of first‐generation feedstock to second‐generation feedstock (e.g., agricultural residues, food waste, etc.).^[^ ^9^, ^10^, ^11^, ^12^, ^13^, ^14^, ^15^ ^]^

Among the promising bio‐based products that can be produced from second‐generation feedstock, PLA succeeded to attract the interest of both the industrial sector and academia in recent years. Some international industrial players have already made large investments in related technologies such as Natureworks, Total Corbion, and ThyssenKrupp Uhde (Uhde Inventa‐Fischer).^[^ ^16^, ^17^, ^18^, ^19^ ^]^ Also, PLA is currently the globally most produced bioplastic with an approximate capacity of around 395 000 tons.^[^ ^20^ ^]^ Moreover, its chemical platform required for PLA production (i.e., lactic acid) is on the list of top ten bioproducts of US department of energy in addition to its high market readiness.^[^ ^21^, ^22^ ^]^

Being biodegradable, recyclable and compostable, the PLA's benefits encompass a variety of applications such as plastic food containers, films, specialty textiles, blends and packaging applications. Moreover, the high biocompatibility of this polymer leads to numerous biomedical applications such as implant devices and tissue scaffolds.^[^ ^23^, ^24^, ^25^, ^26^ ^]^ The wide range of applications is enhanced by its advantageous physical and mechanical properties and better thermal processability compared with other biopolymers (such as PHA, PEG, PCL).^[^ ^27^ ^]^

Not only has PLA an improved sustainability and a wide range of applications, its growing production rate has also the potential for capturing high market shares in the coming decade. According to the forecasts, the investments in PLA production in the United States and Europe are expected to increase up to 2025. At present, the whole bioplastic production comprises only one percent of the total plastic production.^[^ ^28^ ^]^ Therefore, an increased PLA production can contribute to the global transformation from fossil‐based to bio‐based supply chains and the implementation of a circular economy with the already established recycling pathways.^[^ ^29^ ^]^ Hence, PLA is an exemplary sustainable, circular, bio‐based material to be investigated in this study as elaborated in the coming sections.

Many authors have focused on comparing the properties of PLA with the fossil‐based counterparts in order to infer the substitution potentials. Such comparisons included various aspects such as mechanical and thermal properties, processing, etc.^[^ ^30^, ^31^, ^32^, ^33^, ^34^ ^]^ Although these studies have proven the technical compatibility of PLA, no study so far has given a thorough insight on how this can be translated into industrial applications or illustrated the opportunities and challenges of establishing regional supply chains.

According to our best knowledge, there is no interdisciplinary study addressing PLA from technical, economic, and ecological perspectives simultaneously. Hence, the first aim of this study is to review and collect the scattered information on PLA in the literature in a comprehensive manner especially that addresses the second‐generation feedstock. Thereafter, we transform this integrative analysis into a regional model in order to investigate how PLA can flourish in a specific region taking into account the available resources and existing industrial value chains, and determine the knowledge gaps that need to be filled in order to minimize the associated investment risk.

In terms of the region of interest, the German federal state of North Rhine‐Westphalia has been selected for various reasons; first, Germany is one of the largest PLA importers.^[^ ^35^ ^]^ Therefore, the question arises whether it would be preferable and sustainable to produce plastics domestically and thus avoid imports from China or the US. Second, North Rhine‐Westphalia (NRW) is of particular interest as the state is the hub of chemical industry in Germany and Europe; one‐third of the German refining capacities and chemical parks are located in the state.^[^ ^36^, ^37^, ^38^, ^39^ ^]^ Similarly, the chemical sector in NRW earns roughly one‐third of the German chemical industry's turn‐over and employment. Third, the state is witnessing a fundamental structural transformation due to the coal phase‐out, especially in the region of the Rheinisches Revier where half of the German lignite production takes place. According to an economic and structural program, the region should develop from a lignite region into a modern and climate‐friendly energy and industrial region of the future.^[^ ^40^ ^]^ Fourth, the state has various types of second‐generation feedstock that can be used to produce bioplastics (PLA) such as wheat straw and corn stover. Moreover, the potential value chains of producing bioplastics in NRW and Germany are not well covered in the literature.

In terms of structure, the study starts with introducing the main differences between the fossil and bio‐based value chains (Section 2.1). Thereafter, the key technical and environmental aspects of PLA production are discussed. The PLA production processes and suitable feedstock are reviewed in Section 2.2 and the key environmental and recycling aspects are discussed in Sections 2.3 and 2.4. Section 3 focuses on investigating the potentials and challenges associated with establishing PLA supply chains; it starts with introducing the industrial sector in NRW and the major fossil‐based plastics produced in the state. The bio‐based value chains are then presented and the potential PLA production is concluded. Finally, this study presents the main conclusions and highlights the main advantages and drawbacks of establishing a regional bioplastic (PLA) supply chain in NRW (Section 4).

## 2 The Technical and Environmental Aspects of PLA Production

### 2.1 Petrochemical Processes versus Biorefineries

**Figure 1:**
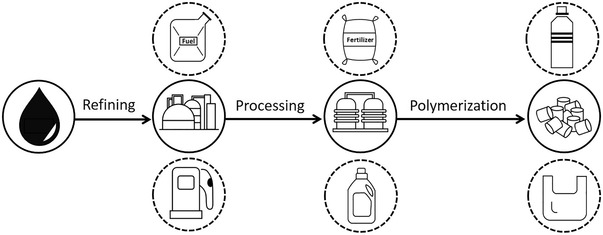
The supply chain of petrochemical industry.

Although the characteristics of fossil‐based and bio‐based products can be comparable, their production processes and value chains are significantly different. As depicted in **Figure** **1**, the supply chain of the petrochemical industry starts with specific inputs (i.e., hydrocarbons such as oil and natural gas) which are processed via refineries into fuels and various intermediate products with different compositions such as ethane, propane, naphtha, etc.^[^ ^41^ ^]^ Some of these products are then processed further to produce various basic chemicals and the required monomers needed for the polymerization process and plastic production.

The sector is highly‐integrated and capital intense, there are roughly 90 refineries and 50 crackers all over Europe.^[^ ^42^, ^43^ ^]^ Moreover, as the points of supply (e.g., oil and natural gas fields, wells, terminals, etc.) and points of demand (i.e., refineries) are concentrated at specific geographical points, big‐scale transportation systems exist. In Germany, there are sophisticated oil and natural gas transportation networks with the required infrastructure in term of ports and railways, terminals, tanks, vessels, etc. This concept is not limited to the primary upstream operations, but the consecutive phases as well. For example, there are already pipeline networks for ethylene and propylene in Germany. Also, there is further consolidation during the production of end‐products such as chemical parks, production clusters, etc.

**Figure 2:**
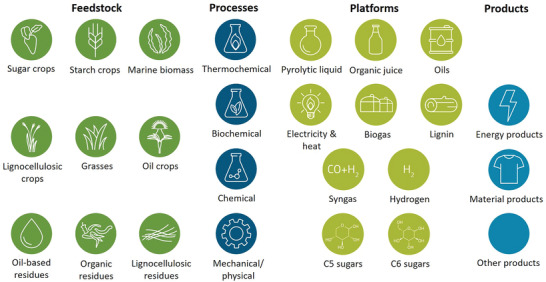
Biorefinery systems (supply chains), based on ref. [44] and translated from ref. [45]. Reproduced with permission, [45] Copyright 2022, BioSC Geschäftsstelle.

On the other hand, the supply chain of biorefineries is more complicated. As shown in **Figure** **2**, a wide range of bio‐resources can be used as a feedstock (e.g., crops, residues, waste, etc.) which are then processed into various platforms (e.g., sugars, lignin, biogas, etc.) via different processes (chemical, thermochemical, etc.) and eventually the final products (e.g., energy, materials, etc.).^[^ ^44^ ^]^ The processes in biorefineries normally do not include a single process; it comprises a complicated series of platforms until the final output is reached.

Although the fossil‐based raw materials (e.g., crude oil) are also not homogeneous as they normally have different density, composition, etc.,^[^ ^46^ ^]^ they are less challenging than bio‐based ones in terms of the consistency of the feedstock. This is already a clear classification for fossil feedstocks and any amount of demand from any type or grade can be sourced without the need to adapt the process to new suppliers, which is not the case in bioeconomy. Moreover, the petrochemical processes and production lines have already witnessed many years of operations, which result in high level of standardization and low‐cost production.

Although the biorefinery is not a novel technology, the wide‐range of potential feedstocks and the evolving concepts aiming at valorizing any available biomass have resulted in many developments in the recent years, which can be manifested in the technology readiness levels of the biorefinery systems. All of these conditions can be considered as hurdles for establishing a regional bioeconomy, especially if the prices of fossil fuels are low or moderate.

### 2.2 PLA Production Process

**Figure 3:**
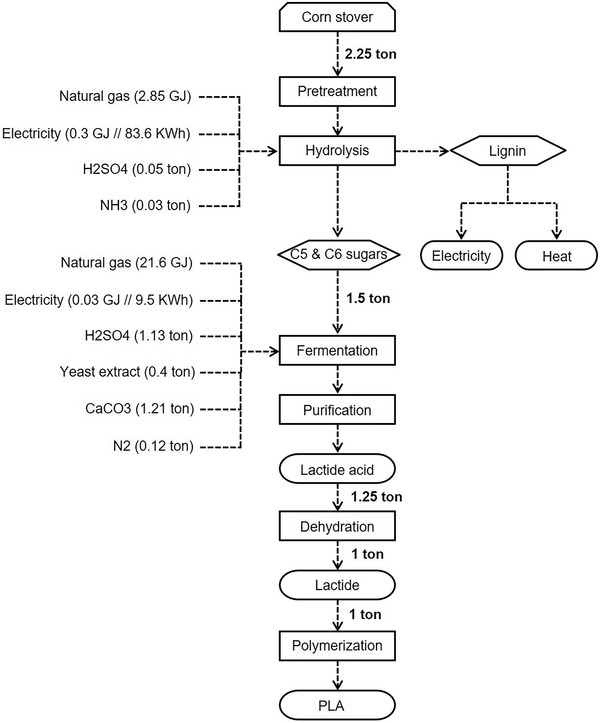
Production process of PLA (corn stover as a feedstock), own visualization based on refs. [22, 48, 49, 50].

PLA is a bioplastic that is both bio‐based and biodegradable. It is made of carbohydrate‐rich raw material from first‐generation feedstock (e.g., corn, wheat or sugar beet) or second‐generation feedstock (e.g., corn stover, wheat straw, etc.). **Figure** **3** shows the example production process of PLA starting from corn stover. After pretreatment and subsequent hydrolysis, C5 and C6 sugars are obtained from the raw material. This procedure is largely the same for other starting materials, whereby extrusion is additionally used in some cases.^[^ ^47^, ^51^ ^]^ In general, a sustainable and efficient sugar production depends on the used biomass (i.e., the starch and carbohydrate amount), the pretreatment conditions and energy input. For example, in the case of corn stover, 1.5 tons of sugar is received from 2.25 tons of raw material.^[^ ^22^ ^]^ For the same amount of sugar, 3.54 tons of wheat are needed.

Various methods to produce sugar from second‐generation feedstock like wheat straw and barley straw have been developed on laboratory scale. However, this is unfortunately not representative for an industrial scale. Momyez et al. showed an environmentally friendly method to produce sugar from wheat straw on a laboratory scale. Via this method, 23.6 g of sugar (17 g glucose and 6.6 g xylose) were obtained starting from 100 g wheat straw through pretreatment, solid preparation, and enzymatic hydrolysis.^[^ ^47^ ^]^ Barley straw biomass can be pretreated on a laboratory scale by different extrusion processes like alkali and enzymatic extrusion to produce fermentable sugars out of it. In 2014, Duque et al. published a yield of 32 g glucose per 100 g dry extrudate and 18 g xylose per 100 g dry extrudate by enzymatic hydrolysis.^[^ ^51^ ^]^ The transformation of the biomass to C5 and C6 sugars enables the production of lactic acid by bacterial fermentation in the next production step.^[^ ^52^ ^]^ Industrially, the lactic acid is transformed to its cyclic dimer lactide, which is the starting material for the bioplastic PLA.

The actual production of PLA is then accomplished in a final polymerization step, so called ring opening polymerization.^[^ ^23^, ^50^, ^53^ ^]^ This mechanism is an established catalytic process for large‐scale PLA synthesis. A tin catalyst is used at industrial scale, which has high activity and leads to profitable properties of the polymer. However, the problem with this catalyst is its toxicity and that the catalyst remains in the polymer, which can lead to an accumulation of toxic residues in the environment.^[^ ^25^, ^54^, ^55^, ^56^ ^]^ Hence, alternative catalysts for industrial use are required. Especially zinc catalysts are suitable substitutes for the industrial complex due to their good biocompatibility, low costs and good activity in this polymerization process. However, not all zinc catalysts are qualified as an alternative. In addition to high activity, the complexes have to be especially robust against impurities and moistures, which is not applicable for all developed catalyst systems.^[^ ^57^ ^]^ Herein, the catalysts ligand design plays an important role. For example, complexes with anionic ligands are sensitive to residues of water and lactic acid presented in the industrially used lactide as well as towards air and moisture. In contrast, neutral donor ligands show robustness towards these impurities and therefore can be used in industrially processes.^[^ ^58^ ^]^ Eco‐toxicology studies confirmed the harmlessness of zinc complexes with neutral donor ligands highlighting their benefits.^[^ ^59^ ^]^ These non‐toxic, industrially relevant catalysts are particularly appropriate for medical applications like implants or drug delivery systems.^[^ ^25^, ^26^ ^]^ In 2020 the fastest known zinc catalyst with a neutral donor ligand system for the polymerization of lactide was published. This catalyst has a higher activity than the industrial used tin catalyst and fulfils the industrial standards of high tolerance to impurities and elevated temperatures, which opens the door for a more sustainable life cycle.^[^ ^60^ ^]^

### 2.3 Life Cycle Assessment

In general, there is an agreement on the environmental advantages of the bio‐based monomer lactic acid (LA) and the polymer PLA in the literature. Similar to other bio‐based products, the storage of biogenic carbon during the feedstock growth decreases the net carbon foot print of LA and PLA in both cradle to gate (Cradle‐to‐gate refers to the product's environmental impact starting from the resource extraction until it is produced) and cradle to grave (Cradle to grave refers to the whole life cycle environmental impact, starting from the resource extraction until disposal (including production and usage).) calculations. This positive carbon credit is not accounted for the conventional plastics as carbon used is sourced via fossil‐based resources. According to Ögmundarson et al.,^[^ ^61^ ^]^ this credit is ≈2.6 kg C_2_O equiv. per ton LA produced from corn, and 2.9 per ton LA produced from corn stover. These numbers are relatively higher than the value reported by Adom et al., 2017,^[^ ^62^ ^]^ which is ≈1.5 per ton LA produced either by corn or corn stover. Such differences can be attributed to the different processes (i.e., lack of standardization), raw materials, system configurations, and boundaries. Hence, the overview presented here shall be considered as guidance or a benchmark rather than a strict environmental evaluation.

**Figure 4:**
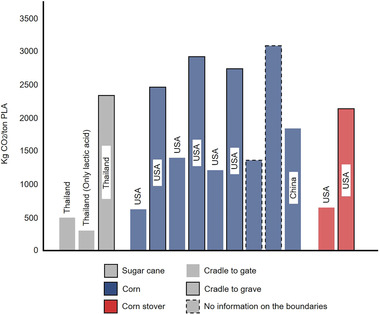
Carbon footprints of PLA production in different regions from different feedstocks.^[^ ^19^, ^62^, ^63^, ^64^, ^65^, ^66^, ^67^ ^]^

**Figure** **4** compares the carbon footprint of PLA production processes in different countries. The cradle‐to‐gate values are generally lower than 1000 kg CO_2_/ton PLA, only two values of 1220 kg CO_2_/ton PLA and 1840 kg CO_2_/ton PLA have been mentioned in Benavides et al., and Zhao et al., respectively.^[^ ^63^, ^64^ ^]^ On the other hand, the cradle‐to‐grave values in the literature are normally higher and range between 2150 kg CO_2_/ton PLA and 2920 kg CO_2_/ton PLA. The difference between cradle‐to‐grave and cradle‐to‐gate highlights the potentials and environmental benefits of recycling, as it will not only help in mitigating the CO_2_ emissions and enable PLA to act as a semi‐permanent carbon sink, but also to increase the PLA amount in order to foster the recycling system.

According to the PLA environmental studies, a major part of the carbon footprint is attributed to the biorefinery activities related to LA production. Herein, the main carbon‐intensive input is the required thermal energy, which is normally sourced via fossil fuels. The relevant studies have also reported various values, as different processes and feedstock can normally result in different energy inputs. Three studies have noted an energy consumption for four processes in a range of (30–40 GJ per ton PLA).^[^ ^19^, ^62^, ^63^ ^]^ Two other studies recorded significantly higher energy inputs for two processes (roughly 60 GJ per on PLA).^[^ ^64^, ^68^ ^]^ Independently of the exact figures, it can be concluded that the current production processes depend on fossil‐based fuels as a cheap and convenient thermal source. Using renewable energies in the future can significantly reduce the carbon footprint and makes PLA a more sustainable product.

### 2.4 PLA Recycling

The recycling of plastic is a key factor to reduce both waste and resource consumption. The high urgency for the implementation of a circular economy, motivated by various EU targets, also demands a significant improvement in plastic recycling in the next years. It is therefore inevitable to judge existing processes with respect to their coherence with a “Renewable Carbon Cycle,” which builds on carbon atoms derived from biomass, air, or existing plastics through recycling. Herein, the energetically most‐convenient solution is the direct recycling of existing plastic. Although PLA is recyclable, it does not enter the conventional recycling system as there is too low amount of PLA material in the post‐consumer plastic mix and, thus, sorting is currently not economically feasible. However, there are existing methods to recycle and compost it and researchers are working on finding further solutions.^[^ ^69^ ^]^

One possible recycling method is mechanical recycling, enabling the production of new products. For PLA this method has the lowest environmental footprint as well as the lowest costs. However, currently it is not certain if it is possible to recycle PLA in the plastic recycling infrastructure due to the contamination of the recycling stream. Moreover, material downgrading is an issue with this method, because it leads to a reuse in less valuable applications. A promising alternative to mechanical recycling is chemical recycling, in which the polymer is broken down to the monomer building blocks. However, the thermal depolymerization of PLA to lactide is complex and expensive. The energy consumption is higher compared to the production of lactic acid by glucose fermentation.^[^ ^70^, ^71^ ^]^ A promising alternative is controlled PLA degradation to value‐added materials such as lactate esters and other platform chemicals, which can be achieved with solvent‐based recycling.^[^ ^29^, ^72^, ^73^ ^]^ A complete recycling of the plastic stock is therefore not possible yet, which requires to fill up material that vanished from the carbon loop by biomass. In this context, not only a huge material loss is observed, but also a yearly loss of $100 billion worth of economic value.^[^ ^74^ ^]^ For other polymers, interesting upcycling options have been explored, for example, from aromatic polymers to arenes,^[^ ^75^ ^]^ or also the carbonization of plastic waste to nanotubes.^[^ ^76^ ^]^ As very recent example for new avenues of upcycling, the aminolysis of PLA to benzyl lactamide was reported.^[^ ^77^ ^]^

Life cycle assessment of different PLA recycling technologies showed lower environmental impacts compared to incineration. In the publication of Maga et al. 2019,^[^ ^78^ ^]^ the global warming impact was determined for different recycling technologies (i.e., mechanical, chemical, and solvent‐based recycling). Their calculations show that mechanical recycling (277 kg CO_2_ equiv. per ton) amounts the least greenhouse gas emissions followed by solvent‐based recycling (521 kg CO_2_ equiv. per ton) and chemical recycling (700 kg CO_2_ equiv. per ton). The authors also included the credit of recyclate produced from PLA waste in their calculation leading to highest potentially saved greenhouse gas emissions of 1519 kg CO_2_ equiv. per ton for solvent‐based recycling (1480 kg CO_2_ equiv. per ton for chemical recycling and 793 kg CO_2_ equiv. per ton for mechanical recycling).^[^ ^78^ ^]^ Considering the substitution of virgin PLA material, it is shown that solvent‐based recycling supports the highest reduction of environmental impacts. However, the other two recycling methods are also suitable for better environmental performance of PLA products. In order to apply these technologies efficiently, the share of PLA waste has to increase. In selected regions, the chemical recycling is already feasible on industrial scale as the example by Total Energies shows.^[^ ^79^ ^]^

### 2.5 Economic Aspects

Economic factors and resource constraints are assumed to be the major barriers toward establishing bioplastics supply chains, or generally a bioeconomy due to several reasons. First, the difference in maturity levels of established fossil‐based and innovative bio‐based technologies results in cost differences of fossil‐based and bio‐based products, that is, bio‐based technologies still have to go through learning curves and economies of scale and scope in implementation. Moreover, there are currently lock‐in effects as fossil‐based technologies are already established and (partly) depreciated, while high upfront investments are needed for new bio‐based technologies. Also, the severe environmental externalities associated with the production and consumption of fossil‐based plastics are still not fully reflected in the prices of fossil‐based plastics (e.g., externalities of GHG emissions).^[^ ^80^, ^81^ ^]^

**Figure 5:**
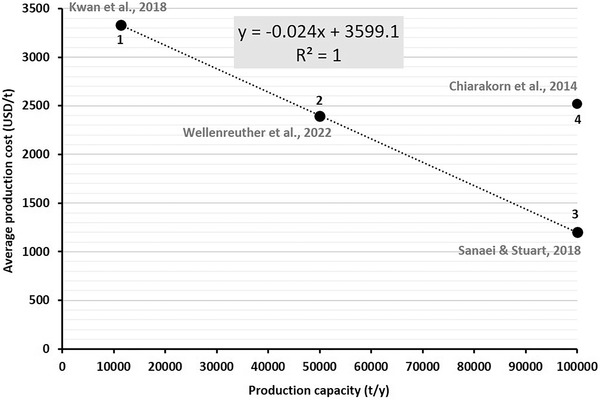
The average production costs versus the production capacity, visualized based on refs. [82, 83, 84, 85].

**Figure 7:**
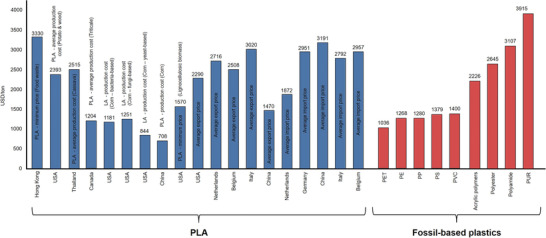
A comparison between the prices of PLA and fossil‐based plastics.^[^ ^49^, ^64^, ^82^, ^83^, ^84^, ^85^, ^86^, ^87^ ^]^

Generally speaking, the PLA production costs are shaped by the investment costs as well as costs for raw materials and energy consumption.^[^ ^82^, ^83^ ^]^ Similar to the environmental impacts of PLA production, there is no consistency in the literature regarding the costs and required investments. **Figure** **5** shows the relationship between the average production costs and the production capacities based on data of four studies.^[^ ^82^, ^83^, ^84^, ^85^ ^]^ As can be seen, the existing analyses in the literature have considered three capacities; approximately 11, 50 and 100 kt per year. As depicted, the first three points on the graph form an ideal linear function. Nonetheless, this clear linear relationship should be presumed with some reservations. First, point 4 is outside the line and even shows higher production costs than point 2, despite having higher capacity. Second, such a linear relation is not common in the capital‐intensive chemical industry as economies of scale normally play a major role. Thus, specific production costs (per ton of product) decrease with increasing capacity. Third, the numbers of ref. [82] are significantly lower than the actual market prices of PLA, as shown later in Figure 7.

Hence, such discrepancies demonstrate the lack of reliable economic data due to dissimilarities in parameters and assumptions, missing data on industrial‐scale processes respectively differing premises on upscaling of lab‐scale processes, as well as a wide range of process configurations and feedstocks. For example, while the analyses of ref. [82] assume Triticale as feedstock, the studies of refs. [84, 85] are based on Cassava and food waste, respectively. Additionally, the mentioned studies represent desk analysis due to the lack of industrial‐scale data, which implies high uncertainties. Similarly, there is a lack of knowledge regarding the costs of PLA recycling. According to our best knowledge, there is no comprehensive study that indicates the cost structure of the recycling process of PLA. Based on the study of ref. [88] which investigates the recycling costs of bioplastics in general, PLA recycling costs in the range of 110–120 EUR per ton could be estimated.

Concluding, the presented preceding discussion on PLA shows that bio‐based production and recycling of this polymer is technically feasible and environmentally advantageous. Therefore, the following chapter focuses on integrating the economic aspects as well as investigating the opportunities and challenges of establishing a regional PLA supply chain.

## 3 Opportunities of Establishing a Regional PLA Supply Chain in NRW

The possibilities of establishing a bio‐based PLA supply chain depend on the regional potentials in terms of the availability of raw materials as well as the existing industrial infrastructure, markets, and consumers. In the following, we analyze if an industrial hub like NRW can be a suitable region to locate a production facility. NRW has been a hub for heavy industries in Germany and Europe. The chemical sector contributes with 13% to the total industrial turnover in the state, which is significantly higher than the other German federal state (e.g., 3% and 4% in Baden Württemberg and Bayern respectively).^[^ ^89^, ^90^, ^91^, ^92^ ^]^ In total, the sector contributes to roughly one‐third of the German chemical industry's turn‐over and employment.^[^ ^36^, ^93^, ^94^ ^]^ Similarly, one‐third of the German refining capacities and chemical parks are located in the state NRW.^[^ ^36^, ^37^, ^38^, ^39^ ^]^

**Figure 6:**
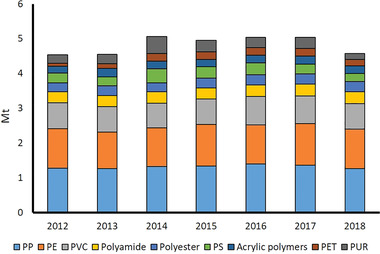
Fossil‐based plastics production in NRW, visualized based on ref. [90].

NRW produces more than 4.5 Mt of nine types of plastics (According to Geyer et al., 2017,^[^ ^96^ ^]^ these plastic types dominate the global plastic market.) (i.e., low‐density and linear low‐density polyethylene, high density polyethylene, polypropylene, polystyrene, polyvinylchloride, polyethylene terephthalate, polyurethanes, polyester, polyamide, and acrylic polymers), of which polyethylene and polypropylene constitute roughly half of the production volume (**Figure** **6**). In terms of market prices, polyethylene, polypropylene, polystyrene, and polyethylene terephthalate have the lowest prices (i.e., 1000–1500 EUR/ton) and polyamide and polyurethanes have the highest prices (i.e., 2500–3500 EUR/ton). As shown in **Figure** **7**, the prices of PLA are generally higher than most of the fossil‐based plastics. Hence, regardless of the technical performance, PLA cannot commercially compete with the plastics with current prices. However, PLA can be converted by the manufacturer into a wide range of products with various applications. Especially in the field of consumer goods, such as the production of home appliances, toys with PLA filaments, PLA fibers for fleeces and faux fur or PLA yarn, the higher price does not necessarily have to be a disadvantage. Consumers may show a higher willingness to pay while making a conscious decision in favor for a more sustainable product.

**Figure 8:**
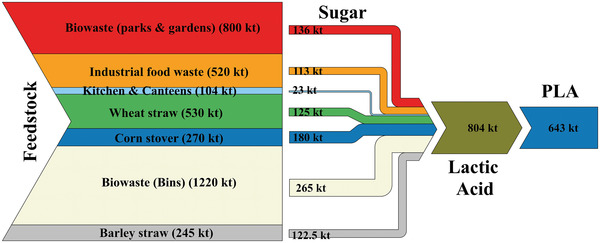
MFA of bio‐resources in NRW, visualized based on refs. [22, 47, 48, 49, 50, 51, 90, 95].

**Figure 9:**
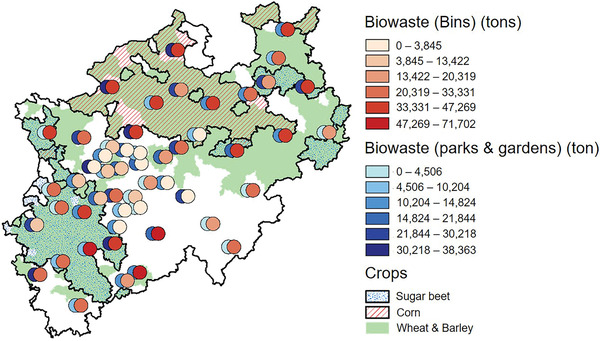
Locations of bio‐resources in NRW, visualized based on ref. [90].

Besides all mentioned aspects, the availability of raw material is a prerequisite for establishing a regional supply chain. Herein, there are extensive amounts of various bio‐resources in NRW which can be used as a feedstock for PLA. In the agriculture sector of NRW, more than one million tons of wheat straw, barley straw and corn stover can be used for bio‐based products.^[^ ^90^ ^]^ The food industry also generates different types of wastes (e.g., sugar production, milk processing, bakery, etc.) which can provide more than half million tons annually. Moreover, the kitchens and canteens generate more than 100 Kt food waste per year. The municipal waste can also supply more than two million tons of biowaste annually.^[^ ^90^ ^]^ Utilizing all of these quantities can provide the required feedstock to produce more than 600 kt PLA annually as shown in **Figure** **8**. However, that should be considered as a theoretical scenario as PLA production will have to compete over the raw materials with other bio‐processes and products. Moreover, there will be various challenges in terms of the homogeneity and quality of all of these different types of materials and wastes. The locational and logistical aspect can be also considered as a major obstacle as the raw materials are disturbed over the state (**Figure** **9**). Although the existing infrastructure can be helpful for feedstock transportation, the transportation and storage costs will be considerable and should be optimized.

## 4 Conclusions and Outlook

PLA is a promising bio‐based and biodegradable bioplastic for a sustainable future. Besides being biodegradable and recyclable, it has the required thermal and mechanical properties to be used for diverse applications. Nonetheless, the discrepancy in the literature regarding the processes, environmental impacts, and costs calls for more research activities in order to yield more reliable and cohesive outcomes. In particular, the focus should be on increasing the TRL, decreasing the production costs and the possibility to include PLA in a circular economy. Herein, the regulations and governmental incentives are mandatory to fill in the technical and economic gap between bio‐based and fossil‐based plastics. The recycling process of PLA should be also investigated further to determine the associated costs and required infrastructure and investments.

Some of discussed challenges could be solved through regional supply chains and production. In terms of the selected region of interest, establishing a regional PLA supply chain in NRW has various opportunities. In addition to the increasing demand due to the consumer shift towards sustainable products, the availability of raw materials and the presence of a robust chemical industry can be considered as strengths. Additionally, the existence of research centers and supporting policies can provide the know‐how and enabling environment for novel bio‐based products to prosper. Nonetheless, these chances are opposed by various challenges that may hinder potential investments such as costs, logistics and the competition over the feedstock resources with other bio‐based products.

## Conflict of Interest

The authors declare no conflict of interest.
